# Preparation and physicochemical characterization of a biodegradable chitosan/carboxymethyl cellulose hydrogel synthesized in NaOH/urea medium

**DOI:** 10.1371/journal.pone.0352207

**Published:** 2026-07-02

**Authors:** Johanna Morales, Allan Vásquez, Irma Patricia Orellana Catalán

**Affiliations:** 1 Departamento de Química, Facultad de Ciencias y Humanidades, Universidad del Valle de Guatemala, Guatemala City, Guatemala; 2 Centro de Estudios en Biotecnología, Instituto de Investigaciones, Universidad del Valle de Guatemala, Guatemala City, Guatemala; University of Waterloo, CANADA

## Abstract

The use of super absorbent polymers in agriculture for water and fertilizers retention in soils has become popular with the increasing need for resource optimization. The objective of the present study was to use chitosan extracted from shrimp shell waste and reagent grade carboxymethylcellulose to synthesize a biodegradable super absorbent polymer with potential use for soil amendment in agriculture. The super absorbent polymer was synthesized using epichlorohydrin as a crosslinking agent in an alkaline NaOH/urea medium. The structure of the product was confirmed by FTIR and TGA. The polymer was found to be biodegradable with a progressive weight loss percentage reaching 79.1% after 14 days. An adsorption ratio of 15.8 and 17.2 was obtained for water and 22% w/v urea solution, respectively, so the product was categorized as super absorbent in both conditions. In addition, after two hours in the medium, absorption percentages of 48.3% were recorded for water and 22% w/v for urea solution. The reported method is effective for synthesizing a biodegradable super absorbent polymer with potential use for soil amendment and susceptibility to pH changes for both adsorption equilibrium and over time adsorption.

## Introduction

Population growth, climate change, urbanization, agricultural expansion, pollution, deforestation, and resource mismanagement are increasing pressures on global water availability. At the same time, freshwater demand continues to rise while accessible resources decline, making efficient water management a strategic priority [1]. Water availability directly impacts multiple development sectors; notably, agriculture accounts for a major share of global freshwater withdrawals and supports a large fraction of food production [2]. These constraints highlight the need for strategies that improve water-use efficiency, enhance crop productivity, and reduce risk under increasingly variable environmental conditions.

Soil amendment refers to the incorporation of materials into soil to improve physical properties and create a more favorable rooting environment. Such improvements may include enhanced water retention, permeability, infiltration, drainage, aeration, and soil structure. Depending on the amendment type and soil conditions, soil amendments can also contribute to reduced mobility of contaminants and decreased transport of agrochemicals into aquatic environments [3]. Overall, the selection of amendment materials is highly context dependent and should be aligned with the intended function and the soil’s specific limitations.

Superabsorbent polymers (SAPs), often referred to as superabsorbent hydrogels, are crosslinked hydrophilic polymer networks capable of absorbing and retaining large amounts of water relative to their dry mass. Their swelling behavior is governed by network structure and by the presence of hydrophilic and ionizable functional groups (e.g., carboxyl and amino groups), and it can be strongly influenced by pH, ionic strength, and other environmental conditions [4–6]. SAPs are commonly synthesized by crosslinking hydrophilic polymer chains through chemical (covalent) crosslinking and/or physical interactions (e.g., hydrogen bonding and ionic interactions) [4,5,7]. Physical approaches such as freeze–thaw cycling can promote network formation by inducing polymer aggregation and stabilizing crosslinking points, with the resulting properties depending on polymer concentration and processing conditions [8].

SAPs can be broadly classified as synthetic, semi-synthetic (hybrid), and natural, based on the origin of their polymeric components. Synthetic SAPs, typically derived from petrochemical monomers, can exhibit high absorbency but may persist in the environment due to limited biodegradability [9–12]. Hybrid systems combine synthetic and natural components to balance performance and degradability, while natural SAPs are based on renewable biopolymers (e.g., cellulose- and chitosan-based materials) and are often valued for biocompatibility and biodegradability [9,13,14]. These attributes motivate continued research into biopolymer-derived SAPs for applications where environmental compatibility is a priority.

The water uptake and release behavior of SAPs is commonly explained by a combination of osmotic pressure and polymer–solvent interactions within the crosslinked network. Upon contact with water or aqueous solutions, hydrophilic and ionizable groups in the polymer network promote solvent ingress. For polyelectrolyte hydrogels, partial ionization of functional groups can generate fixed charges along the polymer backbone, leading to electrostatic repulsion and an osmotic driving force (Donnan effect) that favors swelling. The extent of swelling and subsequent release depends on environmental conditions such as pH and ionic strength, as well as on network crosslink density and composition [9,10,15].

Cellulose is one of the most abundant biopolymers and is widely available from plant-based sources. Chemical modification of cellulose yields a range of functional materials; for example, carboxymethylcellulose (CMC) is a biodegradable and biocompatible derivative of cellulose that has been used to form hydrogels with favorable water retention behavior [11,13,15,16]. Chitin is another abundant natural polysaccharide, commonly obtained from invertebrate exoskeletons [13,17,18]. Chitosan (CS) is a deacetylated derivative of chitin (typically defined by a degree of deacetylation above 50%), and its chemical structure provides amino groups that can contribute to hydrogel formation and pH-responsive behavior [13]. CS-based hydrogels have been widely studied for applications such as biomedical materials, immobilization matrices, controlled delivery systems, and water management materials [19–21].

**Fig 1:**
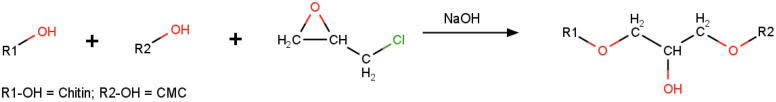
Chitin and CMC crosslinking with ECH [17].

Hydrogels combining chitin/chitosan- and cellulose-derived polymers can be prepared by crosslinking polymer solutions using agents such as epichlorohydrin (ECH) [17,22]. Because chitin is poorly soluble in common solvents, specialized solvent systems have been explored, including ionic liquids, LiCl/dimethylacetamide, and NaOH/urea systems assisted by freeze–thaw processing [5,17,19,20]. In the work by Tang, Chen, Duan, Lu, and Zhang [17], a chitin/CMC hydrogel was synthesized using a NaOH/urea solvent system and ECH as crosslinker (Fig 1). Building on this approach, the present study evaluates the feasibility of preparing a CMC/CS hydrogel under comparable conditions and reports its preliminary physicochemical characterization and water uptake behavior.

**Fig 2:**
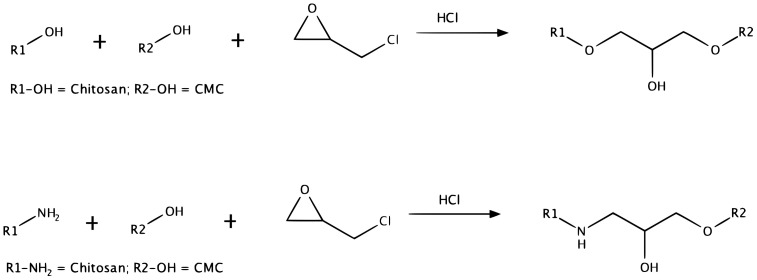
CS and CMC crosslinking with ECH in acid medium [23].

CMC and CS SAPs have also been synthesized with chemical crosslinking [5]. Solvents used include acetic acid for CS and water for CMC, meanwhile some crosslinking agents commonly used are epichlorohydrin (ECH) and glutaraldehyde [23–24]. Acid CS and CMC crosslinking with ECH, done by Wang, Hu, Zhang, Yan, Cui and Zhu [23] shows in Fig 2.

Therefore, the purpose of this study was to use CS extracted from shrimp shells waste, CMC and ECH to synthesize a biodegradable SAP with potential use in soil amendments for agriculture.

## Materials and methods

Reagents used were NaOH (Merck Millipore), urea (HACH), ECH (Aldrich) and CMC (SINOCHEM JIANGSU), chitosan was obtained previously from chitin in shrimp shells by a green method [25]. It´s deacetylation rate was determined to be 82%.

### Chitosan and CMC solution preparation

200mL of NaOH 2.1 M/ and Urea 0.7 M solution were prepared in distillated water. Then 100mL of a chitosan 3% p/v solution were prepared using the NaOH/urea as solvent. A CMC 3%p/v was prepared as well. Both were refrigerated at −20°C for 8 hours and then thawed stirring continuously at room temperature. The freezing/thawing process was done three times with both solutions.

### Chitosan-CMC hydrogel preparation

**Fig 3:**
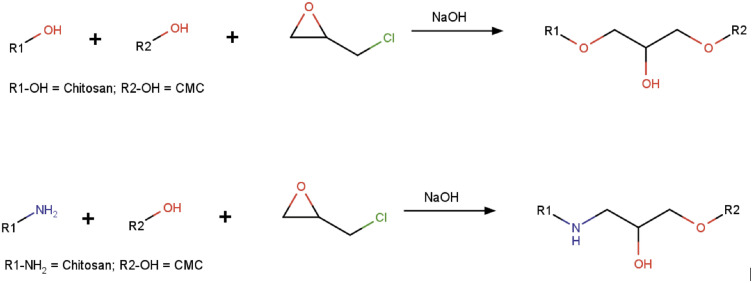
CS and CMC crosslinking with ECH in basic medium.

The chitosan and CMC solutions were mixed at 1:1 proportion. ECH was added as a crosslinking agent. The mixture was agitated at 20°C for 0.5 h and kept at 60°C for 20 min. The reaction conditions are presented in Table 1. The proposed reaction is shown in Fig 3.

**Table 1:** CS/CMC hydrogel synthesis conditions.

CS 3% solution (mL)	CMC 3% solution (mL)	ECH (mL)	Time (min)	T (°C)
100	100	10	20	60

### Characterization

For the SAP characterization FTIR and TGA analysis were carried out. For both analyses a sample of the SAP was dried in an oven at 50°C for approximately 24h. Then, for FTIR analysis, a sample was analyzed using an ATR. For TGA an 8–10 mg sample was introduced to a 20°C/min heating rate from 40°C to 500°C at nitrogen atmospheres. Chitosan and CMC samples were also analyzed in the same conditions.

### Evaluation of biodegradability by soil burial method

SAP was dried in an oven at 50°C for approximately 24h. Then, four samples of around 0.5g each. Four test tubes were prepared with the same amount of soil (pH⁓6.0) and one sample of the weighted SAP was buried in each one at a depth of approximately 5cms. Distillated water was aggregated, and the soil was kept wet for the whole test. Then the SAP samples were removed one by one at different times. The SAP was washed with distilled water and then dried again. Finally, the dried sample was weighted, and the weight loss percentage was calculated using as


$$\begin{array}{c} W_{p}=\left(\frac{w_{i}-w_{f}}{w_{i}}\right)*\text{100} \end{array}$$ (1)

where $W_{p}$ is the weight loss percentage and, $w_{i}$ and $w_{f}$ are the sample´s initial and final weight respectively.

### Swelling measurements

The absorption rate of water and was determined by drying the SAP in an oven at 50°C for approximately 24h, weighting it and then introducing the sample in distillated water. Once swelling equilibrium was reached at room temperature the sample was extracted and weighed. The process was repeated with urea 22% w/v as a fertilizer solution test. Then the absorption rate was calculated as


$$\begin{array}{c} AR=\frac{W_{e}}{W_{s}} \end{array}$$ (2)

where AR is the absorption rate, $W_{e}$ is the hydrogel weight at absorption equilibrium in room temperature and $W_{s}$ is the hydrogel´s weight when dry.

The absorption percentage of both water and urea 22% w/v was determined by using weighted dried SAP and introducing it in the respective medium. Each sample was extracted, weighted and reintroduced in the medium every 5 min for 2h. For each time the absorption percentage was calculated as


$$\begin{array}{c} A_{t}=\frac{W_{t}-W_{s}}{W_{e}}*\text{100} \end{array}$$ (3)

where $A_{t}$ is the absorption and $W_{t}$ is the hydrogel´s weight, both at t time.

## Results

### Freezing/thawing method

A progressive homogenization and gelification was observed for both CMC and chitosan urea/NaOH with each cycle. Both solutions resulted in homogeneous gels.

## Discussion

The dissolution of chitosan and CMC in 2.1 M NaOH/ 0.7 M urea medium demonstrated that the freeze–thawing method is effective for polymer solubilization under the selected conditions. Progressive gelation was observed as the number of freeze–thaw cycles increased, ultimately resulting in homogeneous polymer solutions suitable for subsequent crosslinking.

**Fig 4:**
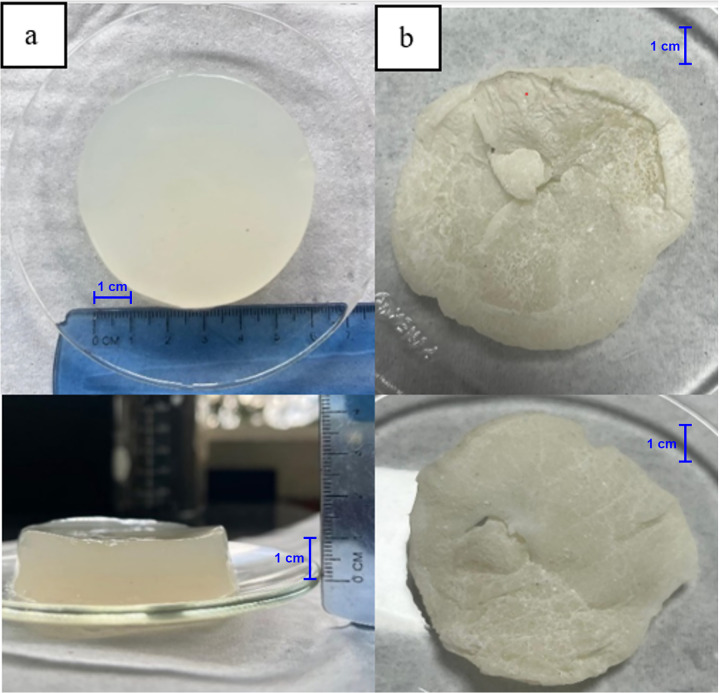
Reaction original and dried product: a original product: Stable, uniform, manipulable and gelified structure and b dried product: porous, reduced, stable and flexible structure.

Following the crosslinking reaction, a stable and uniform hydrogel was obtained. The wet material exhibited a cohesive gel-like structure, while the dried product maintained structural integrity. Descriptive terms related to porosity and mechanical behavior have been moderated, as detailed structural characterization techniques (e.g., SEM, BET, or mechanical testing) were not performed in the present study (Fig 4).

**Fig 5:**
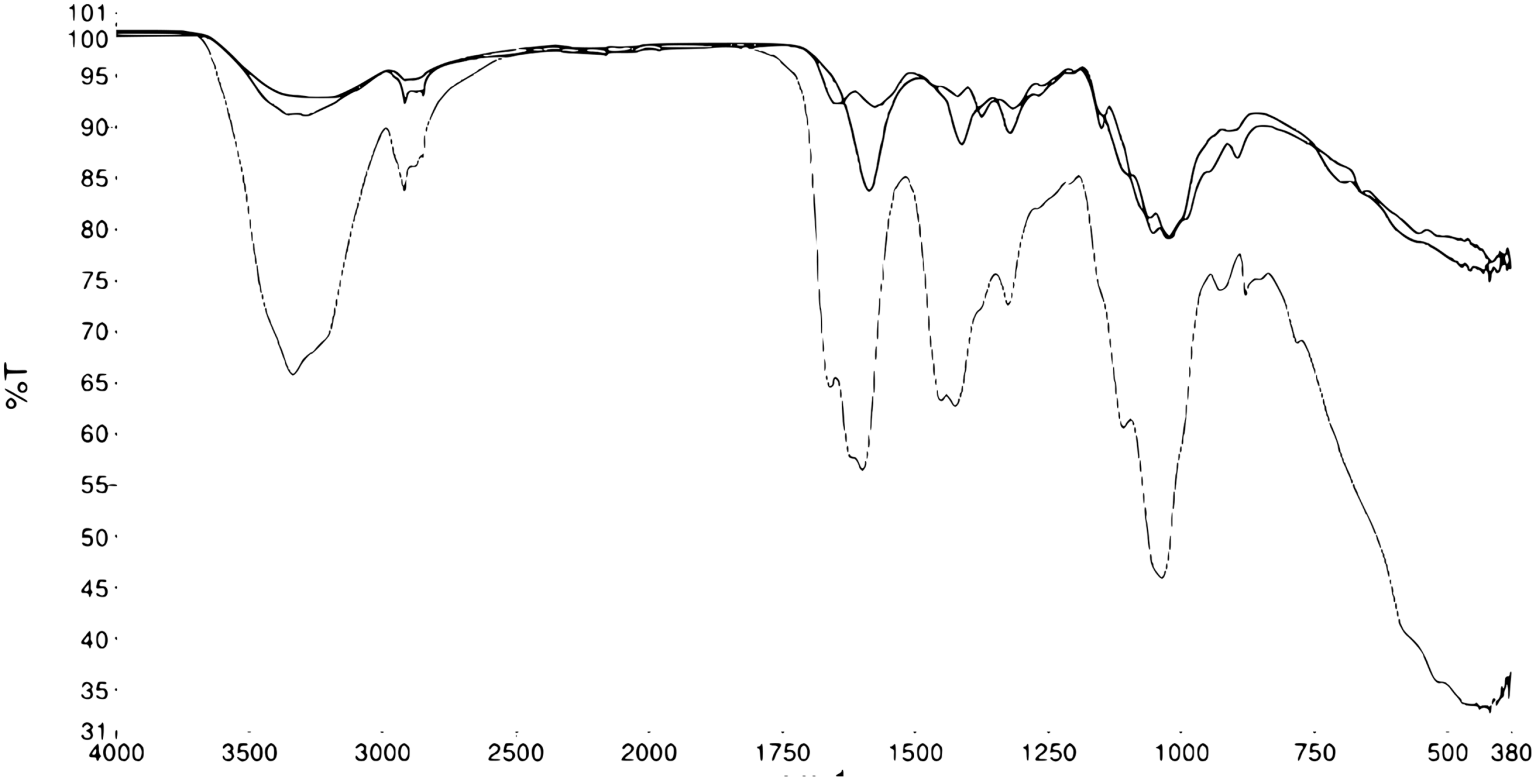
SAP, CS and CMC FTIR spectroscopy: where the results are labeled as Carboximetil celulosa = CMC, SAP quitosano/CMC = product and quitosano = CS.

FTIR analysis (Fig 5) was used to qualitatively evaluate chemical changes associated with hydrogel formation. In the spectra of the resulting polymer, characteristic bands corresponding to chitosan and CMC were observed. In particular, the Amide I and Amide II bands of chitosan were identified, and variations in the intensity and position of bands associated with hydroxyl and ether groups were noted. The broad band around ~3400 cm^−1^ corresponds to overlapping O–H and N–H stretching vibrations, which are present in both starting polymers. Therefore, interpretation of crosslinking was not based solely on this band, but rather on relative spectral changes across multiple characteristic regions. These observations are consistent with previously reported chitosan–CMC hydrogel systems characterized primarily by FTIR analysis [17,24,26].

**Fig 6:**
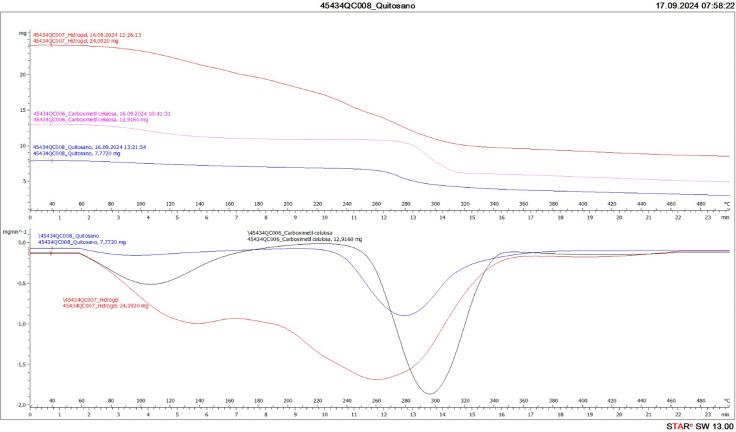
Product, CS and CMC TGA analysis. Where the samples are identified like Hidrogel = Product; Carboximetilcelulosa = CMC and Quitosano = CS.

Thermogravimetric analysis (Fig 6) provided comparative information regarding the thermal behavior of the individual polymers and the synthesized hydrogel. Chitosan exhibited a major weight loss peak near 285 °C, while CMC showed two principal decomposition events around 110 °C and approximately 295 °C. The synthesized hydrogel presented two main weight loss regions: an initial event near 110 °C and a broader degradation range between approximately 200 °C and 310 °C. Rather than confirming chemical structure, these results indicate that the thermal degradation profile of the hydrogel reflects contributions from both polymeric components. The broad degradation region observed for the hydrogel may be attributed to overlapping thermal events within the crosslinked network.

Based on the combined qualitative FTIR analysis and comparative thermal behavior, the formation of a crosslinked chitosan/CMC network in the presence of ECH under alkaline conditions is supported. However, the discussion has been revised to avoid overstating mechanistic details beyond the evidence provided by the employed analytical techniques.

**Fig 7:**
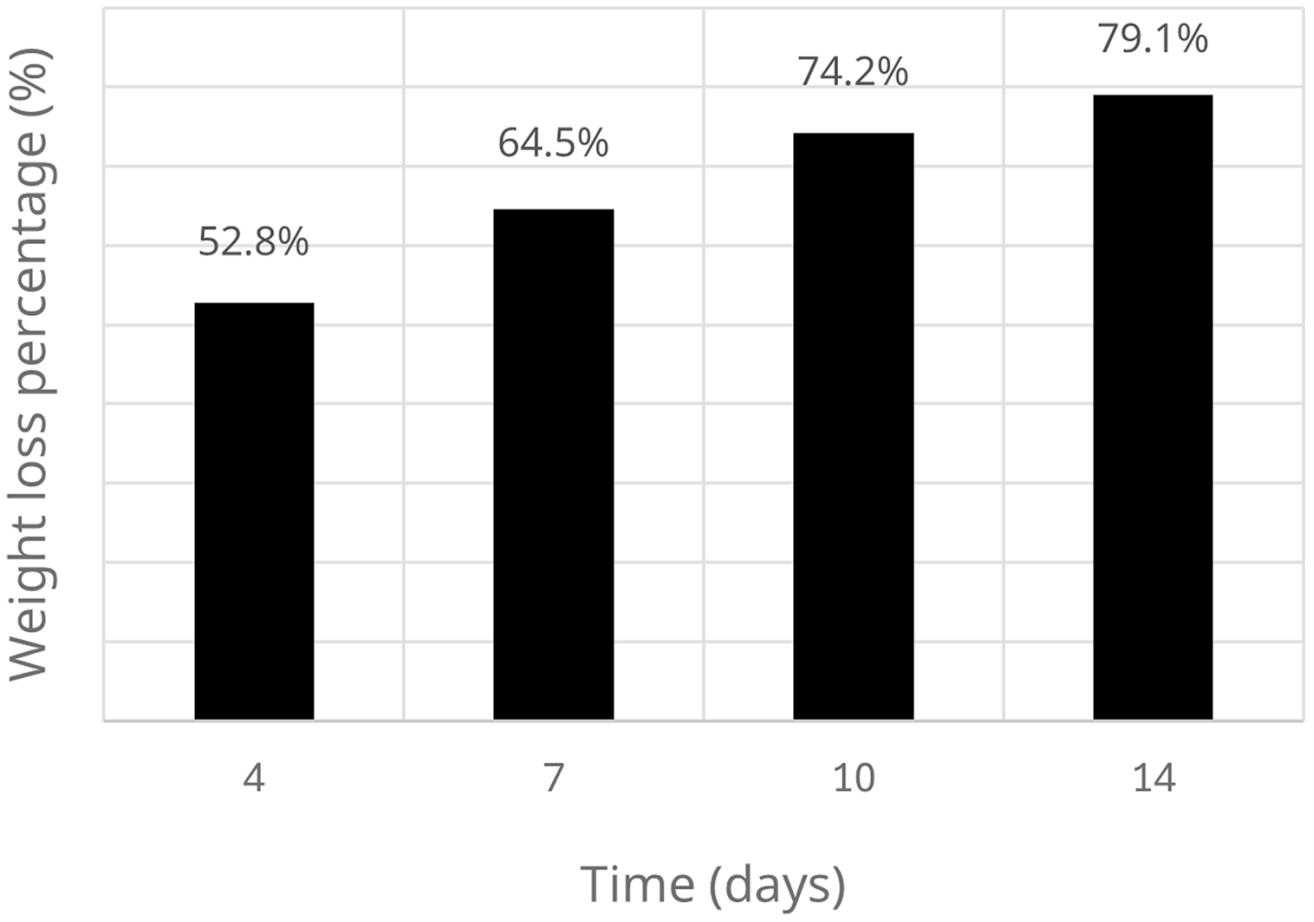
Weight loss percentage.

The hydrogel exhibited progressive mass loss during soil exposure (Fig 7). After four days, approximately 52.8% of the initial mass was lost, and a final mass reduction of 79.1% was recorded after two weeks. Photographic documentation of the material after this period indicates partial structural degradation rather than complete disappearance. Therefore, the degradation observed in this study refers to macroscopic integrity under the specific experimental soil conditions used, and no definitive half-life estimation is proposed.

Regarding swelling performance, the polymer exhibited absorption ratios of 15.8 for water and 17.2 for 22% w/v urea solution (Table 2), values consistent with superabsorbent behavior [6]. The swelling capacity is associated with the presence of hydrophilic functional groups, including carboxyl and hydroxyl groups in CMC and amino and hydroxyl groups in chitosan. Upon contact with aqueous media, partial ionization of these functional groups contributes to osmotic pressure differences and electrostatic repulsion within the polymer network, promoting swelling. The extent of swelling depends on environmental conditions such as pH and ionic strength.

**Table 2:** Hydrogel’s absorption rates of water and urea 22% w/v.

Medium	Absorption rate
Distillated water (pH ⁓7)	15.8
Urea 22% w/v solution (pH ⁓4)	17.2

At mildly acidic pH (~4), chitosan amino groups are predominantly protonated, while carboxyl groups in CMC may be partially ionized. These changes modify the balance of electrostatic interactions within the network and can affect swelling behavior. Nevertheless, water affinity is maintained due to the hydrophilic nature of the polymer chains. In the case of urea solution, hydrogen bonding interactions between urea molecules and functional groups within the hydrogel may contribute to the observed absorption behavior.

In this system, chitosan contributes to network formation and structural integrity within the hydrogel. The role of CMC in enhancing water absorption is consistent with previously reported chitosan–CMC hydrogel systems [17].

**Fig 8:**
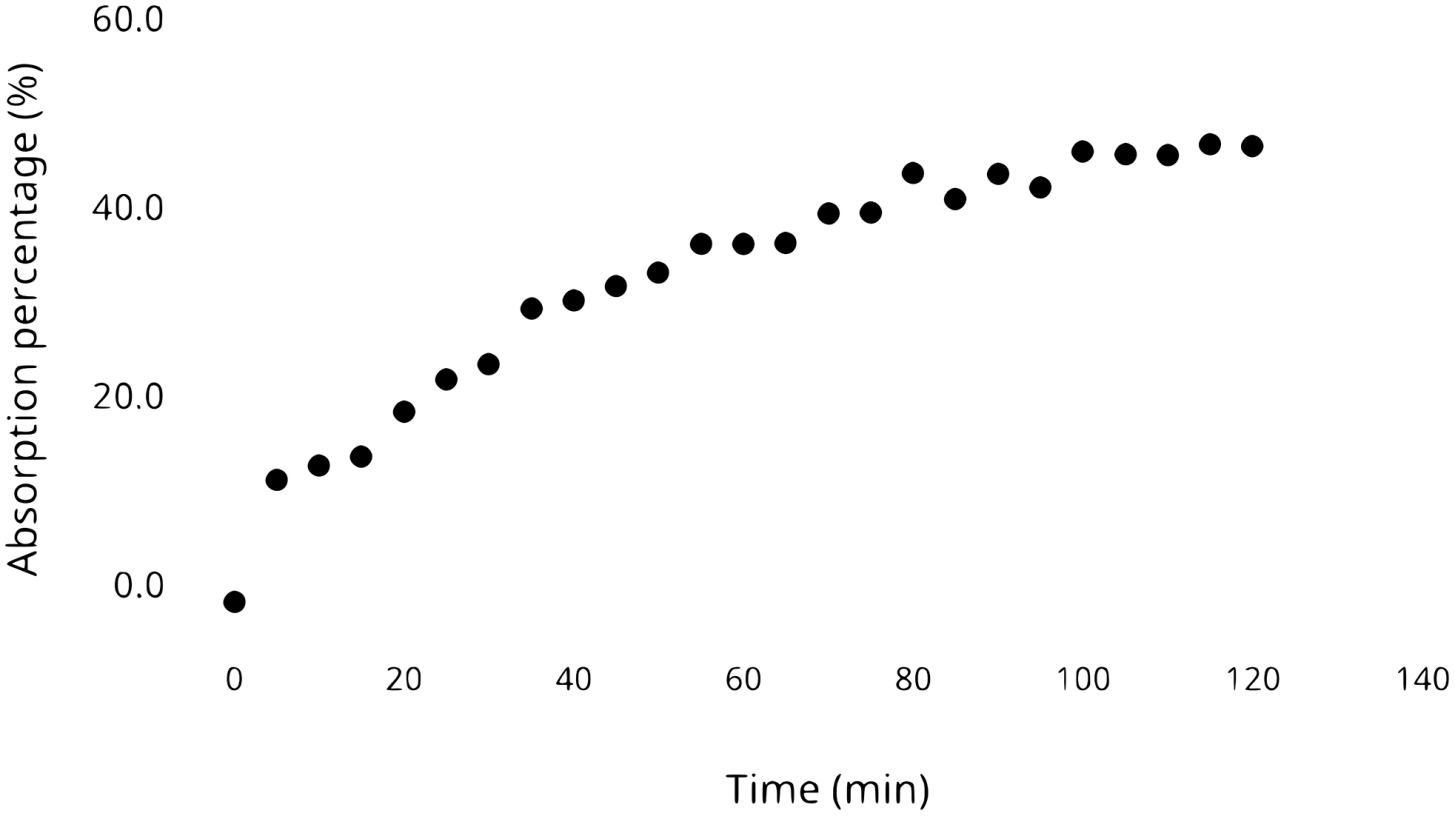
Hydrogel water absorption percentage.

**Fig 9:**
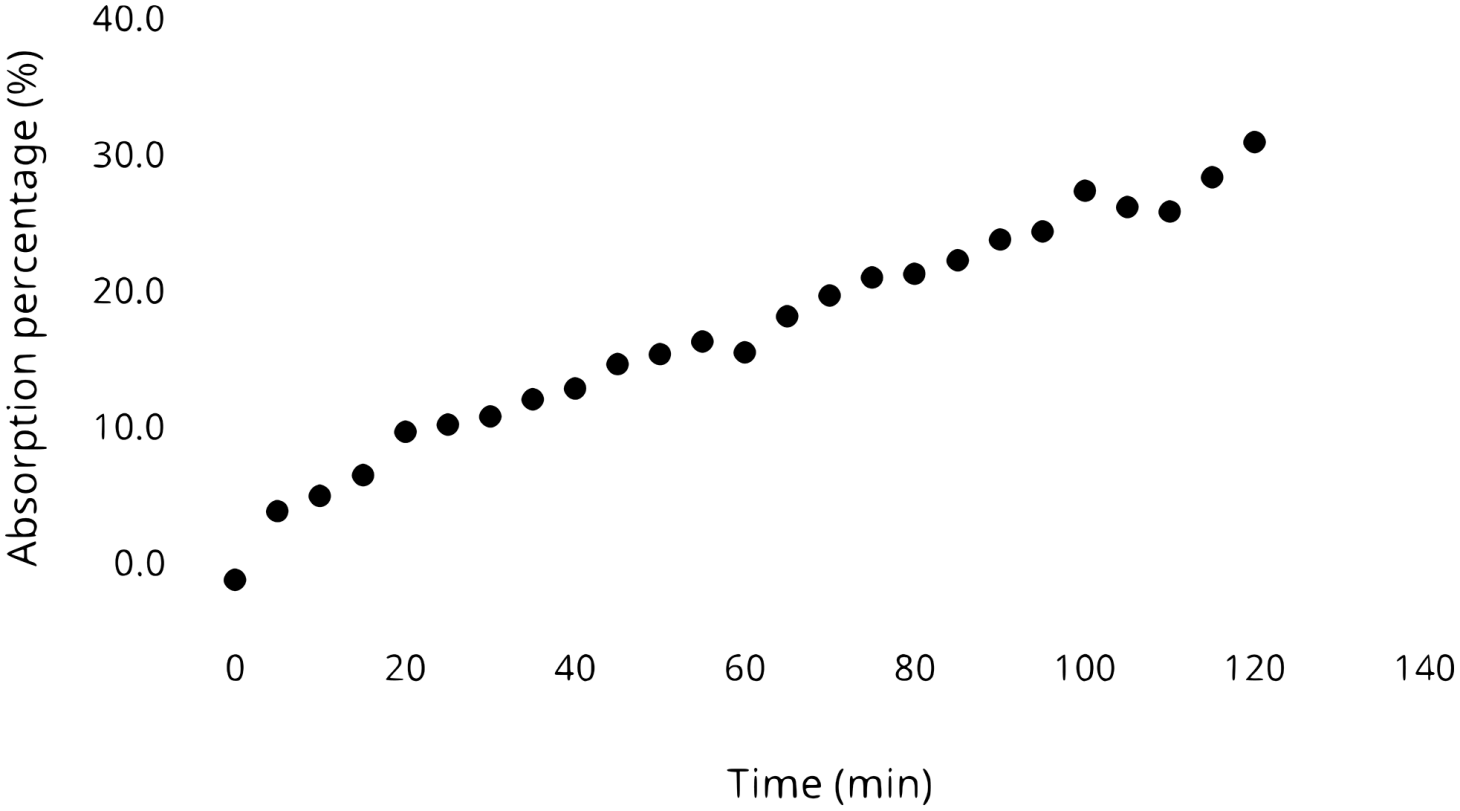
Hydrogel urea 22% w/v absorption percentage.

Finally, the kinetics of solution uptake (Figs 8 and 9) indicate that swelling behavior is influenced by solution composition and pH. Water uptake occurred more rapidly during the first two hours compared to urea solution. Differences in ionic strength and intermolecular interactions may influence short-term swelling rates, while longer-term equilibrium behavior reflects the combined effects of osmotic forces, hydrogen bonding, and network structure. Equilibrium swelling was reached after approximately 26 hours, as reported in the Results section.

Overall, the results demonstrate the feasibility of synthesizing a biodegradable chitosan/CMC hydrogel under the selected conditions and provide preliminary physicochemical characterization and swelling behavior. Further studies incorporating advanced structural characterization and environmental performance testing would be required to comprehensively evaluate its applicability under field conditions.

## Conclusions

This study demonstrated the feasibility of synthesizing a chitosan/carboxymethyl cellulose (CS/CMC) hydrogel in a NaOH/urea system using epichlorohydrin (ECH) as a crosslinking agent. Chitosan extracted from shrimp shell waste and CMC were successfully dissolved through freeze–thaw cycles, enabling the formation of homogeneous polymer solutions suitable for crosslinking. The resulting material exhibited stable gel-like behavior.

Qualitative FTIR analysis revealed characteristic bands associated with functional groups present in chitosan and CMC, and thermogravimetric analysis showed a degradation profile consistent with contributions from both polymeric components. Together, these results support the formation of a crosslinked polymer network under the selected synthesis conditions, within the level of structural evidence provided by the employed analytical techniques.

The hydrogel exhibited progressive mass loss during soil exposure, reaching 79.1% weight reduction after two weeks under the tested experimental conditions. This observation indicates partial biodegradation of the material in soil. Additionally, absorption ratios of 15.8 for water and 17.2 for 22% w/v urea solution demonstrate superabsorbent behavior in both media.

Kinetic analysis showed faster initial water uptake compared to urea solution during the first two hours, while equilibrium swelling was reached at approximately 26 hours. These results indicate that swelling behavior is influenced by solution composition and environmental conditions such as pH.

Overall, the findings provide preliminary physicochemical characterization and swelling performance data for a CS/CMC hydrogel synthesized under alkaline freeze–thaw conditions. Further studies incorporating advanced structural characterization, optimization of synthesis parameters, evaluation under varying environmental conditions, and assessment in realistic soil systems would be necessary to comprehensively determine its long-term environmental performance and practical applicability.

## Supporting information

S1 Fig FTIR spectrum of hydrogel. (PDF)

S2 Fig FTIR spectrum of carboxymethyl cellulose. (PDF)

S3 Fig Overlapping FTIR spectra of hydrogel, carboxymethyl cellulose and chitosan. (PDF)

S4 Fig FTIR spectrum of extracted chitosan. (PDF)

S5 Fig Superimposed FTIR spectra of extracted chitosan and commercial reagent-grade chitosan. (PDF)

S1 Table Absorption ratios by time water:hydrogel and urea:hydrogel. (XLSX)

S2 Table Absorption percentage water:hydrogel and urea:hydrogel. (XLSX)

S3 Table Biodegradability assesment by weight loss percentage. (XLSX)

S4 Table Chitosan FTIR spectra and deacetylation percentage calculations. (XLSX)
