# Supplementary material for: Preparation and physicochemical characterization of a biodegradable chitosan/carboxymethyl cellulose hydrogel synthesized in NaOH/urea medium
Source: PLoS One. 2026 Jul 2;21(7):e0352207. doi: 10.1371/journal.pone.0352207 (PMC13327194; doi:10.1371/journal.pone.0352207)
Supplement: S2 Fig — (PDF) [file pone.0352207.s002.pdf]

Analista Estudiantes UVG

Fecha miércoles, 14 de agosto de 2024 01:16 p.m.

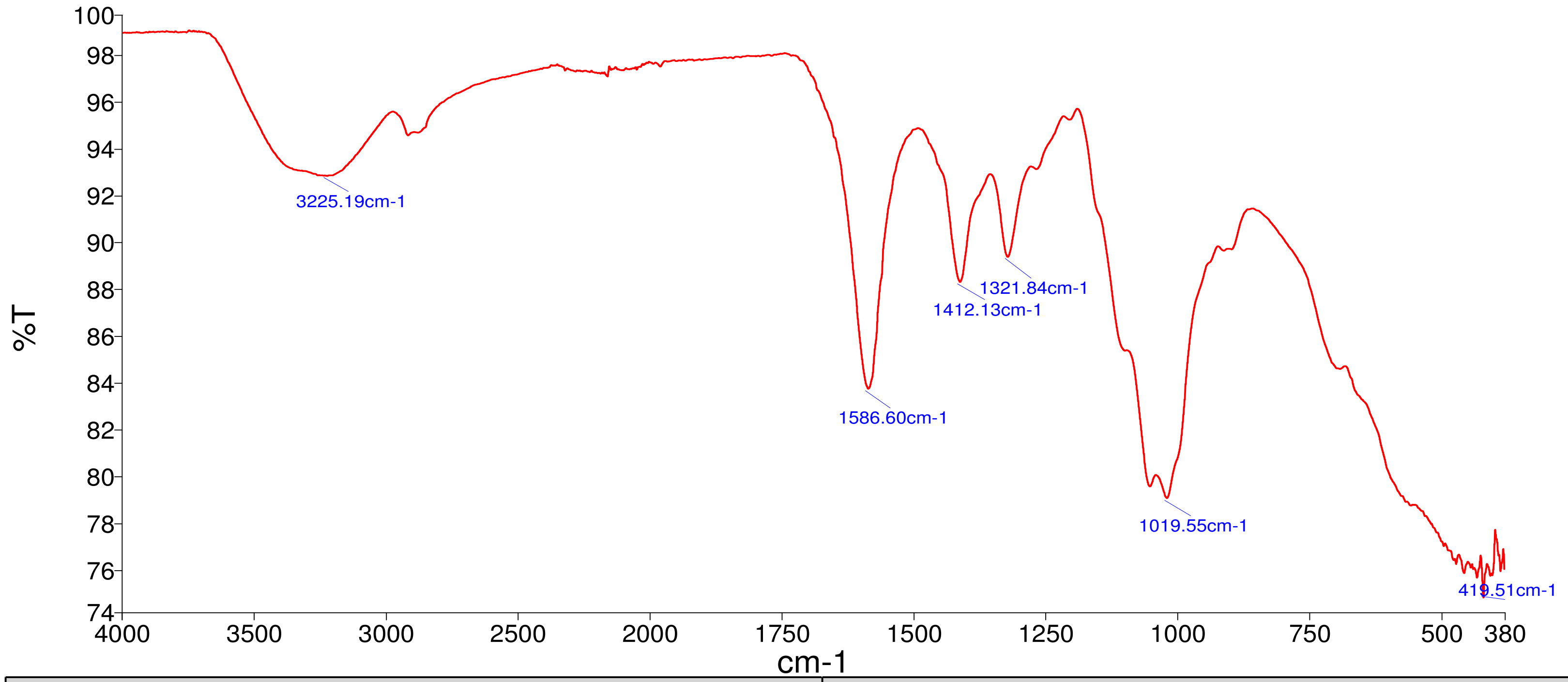

Nombre de la muestra	Descripción
Carboximetil celulosa	Muestra 018 Por Estudiantes Fecha miércoles, agosto 14 2024
