# Supplementary figures and images for: Preparation and physicochemical characterization of a biodegradable chitosan/carboxymethyl cellulose hydrogel synthesized in NaOH/urea medium
Source: PLoS One. 2026 Jul 2;21(7):e0352207. doi: 10.1371/journal.pone.0352207 (PMC13327194; doi:10.1371/journal.pone.0352207)

Analista  
Fecha

Estudiantes UVG  
viernes, 16 de agosto de 2024 01:59 p.m.

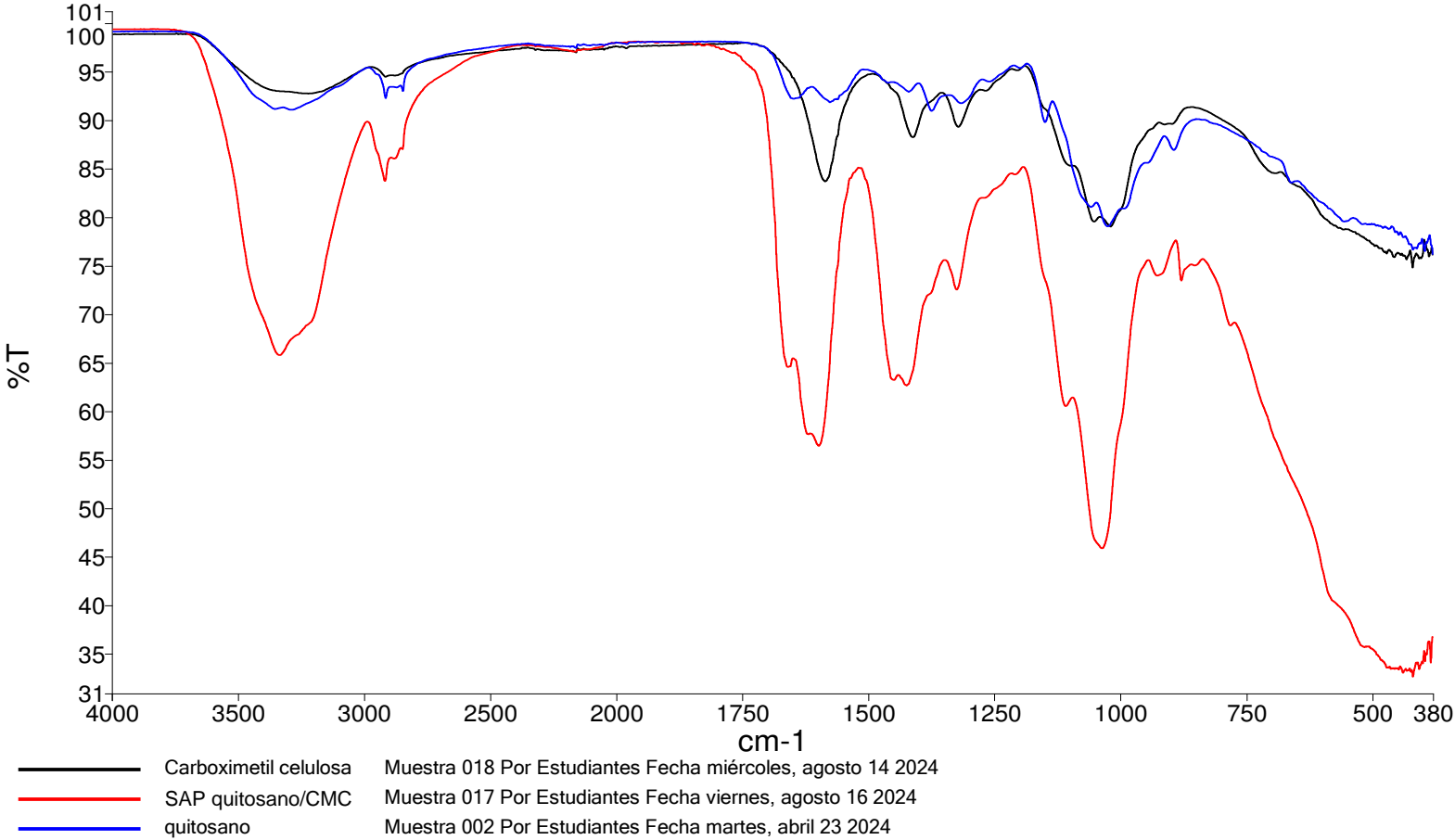

Supplement: S3 Fig — (PDF) [file pone.0352207.s003.pdf]

Analista  
Fecha

Estudiantes UVG  
martes, 23 de abril de 2024 01:21 p.m.

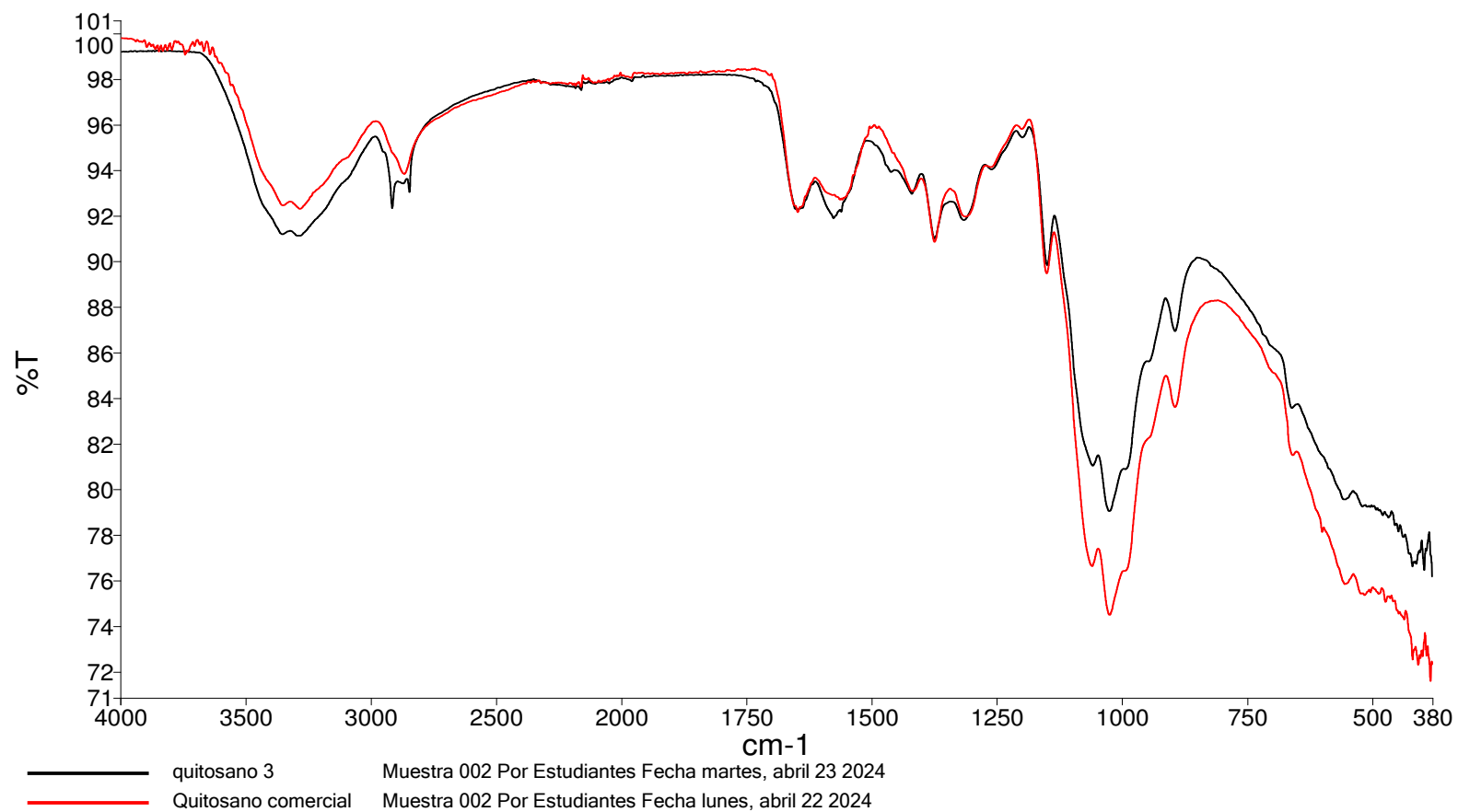

Supplement: S5 Fig — (PDF) [file pone.0352207.s005.pdf]
