# Supplementary material for: Preparation and physicochemical characterization of a biodegradable chitosan/carboxymethyl cellulose hydrogel synthesized in NaOH/urea medium
Source: PLoS One. 2026 Jul 2;21(7):e0352207. doi: 10.1371/journal.pone.0352207 (PMC13327194; doi:10.1371/journal.pone.0352207)

PerkinElmer Spectrum Versión 10.5.4
martes, 23 de abril de 2024 01:20 p.m.

Analista Estudiantes UVG

Fecha martes, 23 de abril de 2024 01:20 p.m.

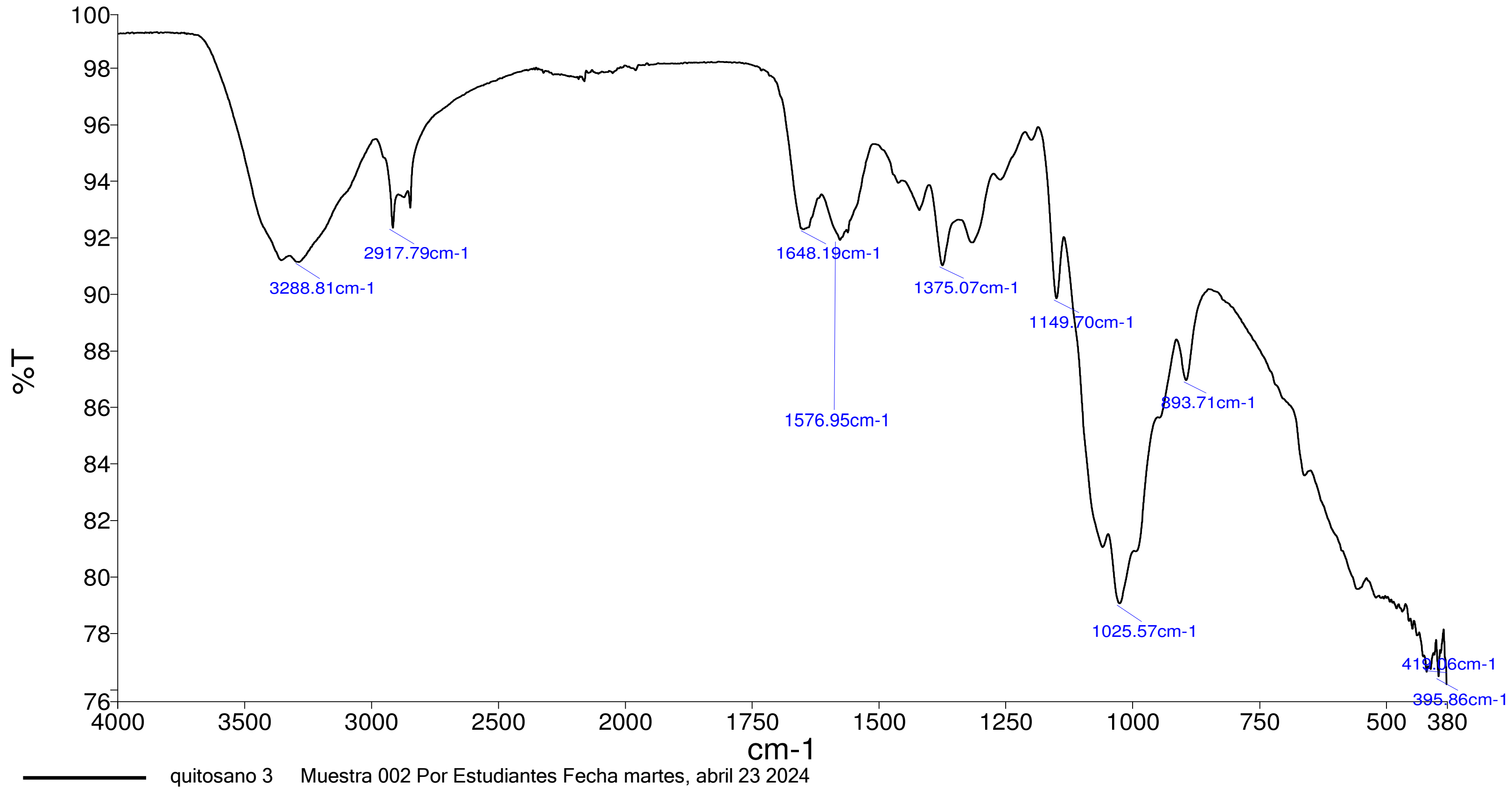

Supplement: S4 Fig — (PDF) [file pone.0352207.s004.pdf]
